# Contrasting Population Structures of Two Vectors of African Trypanosomoses in Burkina Faso: Consequences for Control

**DOI:** 10.1371/journal.pntd.0001217

**Published:** 2011-06-28

**Authors:** Naférima Koné, Jérémy Bouyer, Sophie Ravel, Marc J. B. Vreysen, Kouadjo T. Domagni, Sandrine Causse, Philippe Solano, Thierry de Meeûs

**Affiliations:** 1 Unité de Formation et de Recherche Biosciences, University of Abidjan, Abidjan, Ivory Coast; 2 Centre International en Recherche-Développement sur l'Elevage en Zone Subhumide (CIRDES), Bobo-Dioulasso, Burkina Faso; 3 Cirad, UMR CIRAD-INRA Contrôle des Maladies Animales Exotiques et Émergentes, Montpellier, France; 4 Isra-Lnerv, Service de Parasitologie, Dakar-Hann, Sénégal; 5 Institut de Recherche pour le Développement (IRD), UMR177 IRD-CIRAD, Montpellier, France; 6 Insect Pest Control Laboratory, Joint FAO/IAEA Programme of Nuclear Techniques in Food and Agriculture, Vienna, Austria; 7 UEMOA, Département du Développement Rural, des Ressources Naturelles et de l'Environnement (DDRE), Direction des Ressources Animales et Halieutiques (DRAH), Ouagadougou, Burkina Faso; 8 CNRS, Délégation Languedoc-Roussillon, Montpellier, France; Foundation for Innovative New Diagnostics (FIND), Switzerland

## Abstract

**Background:**

African animal trypanosomosis is a major obstacle to the development of more efficient and sustainable livestock production systems in West Africa. Riverine tsetse species such as *Glossina palpalis gambiensis* Vanderplank and *Glossina tachinoides* Westwood are the major vectors. A wide variety of control tactics is available to manage these vectors, but their removal will in most cases only be sustainable if the control effort is targeting an entire tsetse population within a circumscribed area.

**Methodology/Principal Findings:**

In the present study, genetic variation at microsatellite DNA loci was used to examine the population structure of *G. p. gambiensis* and *G. tachinoides* inhabiting four adjacent river basins in Burkina Faso, i.e. the Mouhoun, the Comoé, the Niger and the Sissili River Basins. Isolation by distance was significant for both species across river basins, and dispersal of *G. tachinoides* was ∼3 times higher than that of *G. p. gambiensis*. Thus, the data presented indicate that no strong barriers to gene flow exists between riverine tsetse populations in adjacent river basins, especially so for *G. tachinoides*.

**Conclusions/Significance:**

Therefore, potential re-invasion of flies from adjacent river basins will have to be prevented by establishing buffer zones between the Mouhoun and the other river basin(s), in the framework of the PATTEC (Pan African Tsetse and Trypanosomosis Eradication Campaign) eradication project that is presently targeting the northern part of the Mouhoun River Basin. We argue that these genetic analyses should always be part of the baseline data collection before any tsetse control project is initiated.

## Author Summary

Tsetse flies are insects that transmit trypanosomes to humans (sleeping sickness) and animals (nagana). Controlling these vectors is a very efficient way to control these diseases. In Burkina Faso, a tsetse eradication campaign is presently targeting the northern part of the Mouhoun River Basin. To attain this objective, the approach has to be area-wide, i.e. the control effort targets an entire pest population within a circumscribed area. To assess the level of this isolation, we studied the genetic structure of *Glossina palpalis gambiensis* and *Glossina tachinoides* populations in the target area and in the adjacent river basins of the Comoé, the Niger and the Sissili River Basins. Our results suggest an absence of strong genetic isolation of the target populations. We therefore recommend establishing permanent buffer zones between the Mouhoun and the other river basin(s) to prevent reinvasion. This kind of study may be extended to other areas on other tsetse species.

## Introduction

Tsetse flies (Diptera: Glossinidae) are the sole cyclical vectors of human and animal trypanosomoses, two major plagues that are seriously impeding African development. African animal trypanosomosis (AAT) is a major obstacle to the development of more efficient and sustainable livestock production systems in West Africa. Since 2008, the Government of Burkina Faso has embarked on an ambitious tsetse eradication campaign that targets the northern Mouhoun River Basin for its first phase (http://www.pattec.bf/). The Mouhoun River Basin eradication campaign is implemented under the auspices of the Pan African Tsetse and Trypanosomosis Eradication Campaign (PATTEC), an African Union initiative that was launched in 2001 following an historic decision by the African Heads of State and Government in Lome, Togo, July 2000 (http://www.africa-union.org/Structure_of_the_Commission/depPattec.htm).

In the Mouhoun River Basin, *Glossina palpalis gambiensis* Vanderplank and *Glossina tachinoides* Westwood are the two remaining tsetse species, after the regression of *Glossina morsitans submorsitans* Newstead [1]–[3]. The two tsetse species remain very effective vectors of AAT [4], but local transmission of sleeping sickness (Human African Trypanosomosis (HAT)) seems to have disappeared from the Mouhoun River Basin [3]. These species inhabit the riparian forests that form habitat galleries along the rivers and the flies' relative abundance is determined by forest ecotype and its level of fragmentation and destruction [2], [5]. Their particular resilience to habitat fragmentation has been attributed to (1) their ability to easily adapt to peridomestic situations, (2) their opportunistic host feeding behaviour [6], and (3) their linear habitat that allows them to easily disperse between favourable patches, i.e. riverine forests acting as “genetic corridors” [7], [8].

Control of tsetse can be achieved through a variety of techniques [9], including traps, insecticide-impregnated targets [10], live-baits [11]–[13], sequential aerosol technique [14], and the sterile insect technique (SIT) [15]. In the past, most control efforts were not sustainable due to either flies surviving the initial interventions, or flies immigrating from untreated regions, or both [16]. The strategic choice between eradication and suppression of a tsetse population is of prime importance as it will have significant economic implications (see [17] for a review). In that respect, knowledge of the genetic structure of the target population can facilitate this critical decision making [18]–[20]. For isolated tsetse populations, eradication is undoubtedly the most cost-effective strategy, as was demonstrated with the sustainable removal of *Glossina austeni* Newstead from the Island of Unguja, Zanzibar in 1994–1997 [15]. On mainland Africa, the geographical distribution limits of the target tsetse populations are less clearly defined, although complete isolation was recently demonstrated for a *G. p. gambiensis* population in the Niayes area of Senegal that prompted the Government of Senegal to select an eradication strategy [20], [21].

In Burkina Faso, *G. p. gambiensis* populations inhabiting fragmented habitats are genetically structured along the rivers [22], also in the area that is the target of the national eradication campaign mentioned above. However, a certain level of gene exchange is still sustained among the various populations that inhabit the habitat fragments along the Mouhoun River. Furthermore, *G. tachinoides* occurs as a panmictic population along its riverine habitat in the same area, due to its more xerophylous nature allowing it to disperse more easily between suitable habitat patches [23]–[25]. As riverine tsetse populations are mainly confined to the riverbeds of the various river systems which are organised in river basins it was proposed to use the “river basin” as a unit of operation in area-wide integrated pest management (AW-IPM) programmes [28] against tsetse in West Africa. This assumed that each primary river basin (and possibly also secondary and tertiary) contained riverine tsetse populations that were geographically isolated from those belonging to adjacent river basins. If this hypothesis proves to be correct, it would be very beneficial for the present eradication campaign since it would allow limiting the control effort to the Mouhoun River Basin. However, earlier studies have indicated that riverine tsetse flies were able to disperse up to 2km into the savannah areas bordering the riparian forests [7] and a recent genetic study in Burkina Faso suggested that *G. p. gambiensis* was able to cross the watershed divide between the Mouhoun and the Comoe river basins that contained natural woody savannah [26]. In view of the importance of the Mouhoun eradication project, and the limited number of samples (three) used in previous study [26], it was deemed necessary to expand these studies and to obtain more data on the dispersal potential of the two tsetse species present, as evidenced through genetic structures of the various populations. A more complete picture of the exchange of genes between the various tsetse populations in the area would enable the programme managers to make informed decisions on the establishment of buffer zones between the Mouhoun River Basin and its neighbouring basins, or, alternatively, to expand the eradication campaign to these basins.

**Figure 1:**
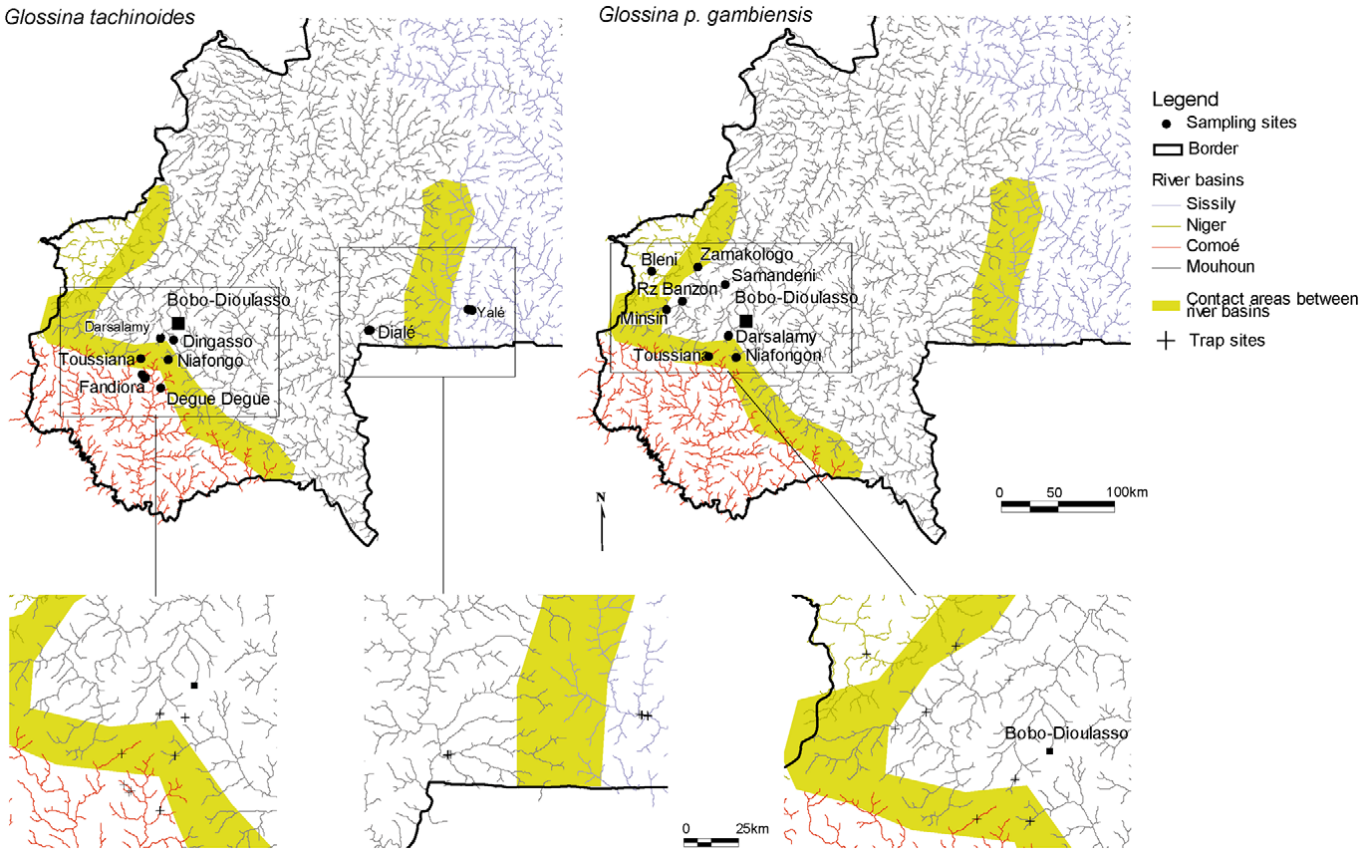
Location of the sampling sites, rivers basins and buffer areas between these river basins.

The present study includes *G. tachinoides* and two other river basins not considered earlier and also includes areas where the interfluve is very much fragmented, which might impact dispersal of riverine species. Genetic variation at microsatellite DNA loci was thus used to examine the structure of *G. p. gambiensis* and *G. tachinoides* populations of the Mouhoun River Basin in relation to those of all its adjacent river basins, i.e. the Niger (Bani), Comoé and Sissili River Basins (Figure 1). The objective was to assess tsetse population structuring in and between the different river basins, its relation to tsetse fly dispersal amongst adjacent river basins, and its consequences for potential AW-IPM eradication campaigns [27], [28].

## Materials and Methods

### Study Site

The study area is located in South-Western Burkina Faso (latitude 10.2 to 12.2 N; longitude −5.5 to −2.0°W) and encompassed the Mouhoun River Basin (8 sampling sites) and three neighbouring river basins, i.e. the Comoe (3 sampling sites), the Sissili and the Niger (1 sampling site each) River Basins (fig. 1). From November 2007 to March 2008 each site was sampled using 5–10 unbaited biconical traps [29]. In each location, the maximal river length sampled was 980 m (in Darsalamy) for *G. p. gambiensis* and 5660 m for *G. tachinoides* (Fandiora), but was usually lower than 500 m (Tables 1&2).

**Table 1:** Number of *G. tachinoides* genotyped in each site and description of the sampling system.

River basin	Site	Females	Males	Total	Number of trap sites	Mean distance between trap sites	Total river length sampled
Comoe	Degue Degue	18	17	35	3	212	424
	Fandiora	16	19	35	3	2830	5660
	Toussiana	25	10	35	2	210	210
Mouhoun	Darsalamy	24	10	34	4	327	980
	Dialé	36	20	56	9	264	2115
	Dingasso	19	20	39	5	70	280
	Niafongo	17	25	42	4	130	391
Sissili	Yalé	15	5	20	9	540	4316
All		170	126	296	39	464	14376

**Table 2:** Number of *G. p. gambiensis* genotyped in each site and description of the sampling system.

River basin	Site	Females	Males	Total	Number of trap sites	Mean distance between trap sites (m)	Total river length sampled (m)
Comoe	Toussiana	12	12	24	2	210	210
Mouhoun	Darsalamy	18	15	33	4	320	960
	Minsin(pindia)	15	11	26	4	110	330
	Niafongon	13	15	28	4	135	404
	Rz banzon	20	10	30	6	88.2	441
	Samandeni	24	12	36	2	73	73
	Zamakologo	23	12	35	3	145	290
Niger	Bleni	20	10	30	6	79	395
All		145	97	242	31	135	3103

### Sampling and genotyping

A total of 296 *G. tachinoides* and 242 *G. p. gambiensis* flies were genotyped (see number of flies genotyped by trapping site in Tables 1&2). *G. p. gambiensis* was genotyped at 8 microsatellite loci: Gpg 55.3 [30], A10, B104, B110, C102 (kindly supplied by A. Robinson, Insect Pest Control Laboratory (formerly Entomology Unit), Food and Agricultural Organization of the United Nations/International Atomic Energy Agency [FAO/IAEA], Agriculture and Biotechnology Laboratories, Seibersdorf, Austria), pGp13, pGp24 [31], and GpCAG [32]. *G. tachinoides* was genotyped at 9 microsatellite loci: pGp13, pGp17, pGp20, pGp24, pGp28, pGp29 [31], B104, C102 and GpCAG. Of these, B104, B110, pGp13, pGp20, and 55.3 are known to be located on the X chromosome. GpCAG and C102 have trinucleotide repeats whereas the others are dinucleotides.

Three legs of each individual tsetse fly were removed, transferred to a tube to which 200 µl of 5% Chelex chelating resin was added [33], [34]. After incubation at 56°C for one hour, DNA was denatured at 95°C for 30 min. The tubes were then centrifuged at 12,000 g for two min and frozen for later analysis. The PCR reactions were carried out in a thermocycler (MJ Research, Cambridge, UK) 20 µl final volume, using 10 µl of the diluted supernatant from the extraction step as template. After PCR amplification, allele bands were routinely resolved on a 4300 DNA Analysis System from LI-COR (Lincoln, NE) after migration in 96-lane reloadable (3x) 6.5% denaturing polyacrylamide gels. This method allows multiplexing by the use of two infrared dyes (IRDye), separated by 100 nm (700 and 800 nm), and read by a two channel detection system that uses two separate lasers and detectors to eliminate errors due to fluorescence overlap. To determine the different allele sizes, a large panel of about 70 size markers was used. These size markers had been previously generated for *G. p. gambiensis* by cloning alleles from individual tsetse flies into pGEM-T Easy Vector (Promega Corporation, Madison, WI, USA), but were generated for *G. tachinoides* for this study. Three clones of each allele were sequenced using the T7 primer and the Big Dye Terminator Cycle Sequencing Ready Reaction Kit (PE Applied Biosystems, Foster City, CA, USA). Sequences were analyzed on a PE Applied Biosystems 310 automatic DNA sequencer (PE Applied Biosystems) and the exact size of each cloned allele was determined. PCR products from these cloned alleles were run in the same acrylamide gel as the samples, allowing the allele size of the samples to be determined accurately [35]. The gels were read twice by two independent readers using the LIC-OR Saga genotyping software.

### Data Analyses

All datasets were processed with Create V 1.1 [36] and converted into the appropriate format as needed.

Wright's *F*-statistics [37] were estimated with Weir and Cockerham's unbiased estimators [38] under Fstat V 2.9.4 (Goudet 2003, updated from [39]). *F*_IS_ is a measure of local inbreeding of individuals relative to inbreeding of subsamples. It is therefore also a measure of reproductive strategy and varies from -1 (all individuals are heterozygous for the same two alleles within each subsample) to +1 (all individuals are homozygous with at least two alleles in subsamples) and equals 0 when all subsamples conform to genotypic proportions expected under panmixia. It is thus also a measure of deviation from the random mating model within populations. *F*_ST_ measures inbreeding of subsamples relative to the total inbreeding resulting from subdivision. It is therefore also a measure of differentiation among subsamples. It varies between 0 (no differentiation) and 1 (all subsamples fixed for one or the other allele).

The significant departure from 0 of these parameter estimates was tested by randomisation procedures under Fstat. For this, alleles are randomly exchanged between individuals in each subsample and the proportion of times when a *F*_IS_ estimate was equal to or higher than the observed one provided the exact *P*-value of the test. For differentiation between populations, individual were randomised across subsamples and the statistic used here was the log-likelihood ratio *G* as recommended [40].

Linkage disequilibrium (LD) between loci was also tested through randomising association between each locus pair. For each pair of loci the tests were combined across subsamples with the *G*-based procedure as recommended [41]. All these randomisations (10000 in each case) were undertaken with Fstat 2.9.4.

For LD, there were as many tests as there were loci pairs (here possibly 36), we therefore tested the probability of obtaining a proportion higher than the expected one (5%) with a binomial test with *k* tests, mean 0.05 and *k_s_* success (the number of significant pair in linkage disequilibrium at level α = 0.05) with MultiTest V 1.2 [41].

More than three levels (i.e. individuals, sub-populations and total) exist within the samples of each tsetse species. Individuals were caught in different traps, in different sites (i.e. locations) within three different river basins (Comoé, Mouhoun and Sissili for *G. tachinoides* and Comoé, Mouhoun and Niger for *G. p. gambiensis*). Hierfstat version 0.03–2 [42] is a package for the statistical software R. This package computes hierarchical *F*-statistics from any number of hierarchical levels [42]. *F*_Trap/Site_ represents the homozygosity due to the subdivision into different traps in each site and was tested by randomising individuals between traps within each site. *F*_Site/Basin_ represents the homozygosity due to subdivision into different sites within each river basin and was tested by randomizing traps (with all individuals contained) between sites within the same river basin. *F*_Basin/Total_ measures the relative homozygosity due to the geographical separation between river basins and was tested by randomizing sites (with all traps included) between the three river basins. In all cases we undertook 1000 permutations and the log likelihood ratio as for the *F*_ST_ analysis was the statistic used. These tests were performed with Hierfstat. A user friendly step by step tutorial of how to use HierFstat is available [43].

Some microsatellite loci, noted with an X as last letter, are X linked. These loci were coded as missing data for *F*_IS_ and null allele analyses and coded as homozygous for the allele present on the X for differentiation and LD tests.

Significant *F*_IS_ can be due to null alleles, stuttering or short allele dominance. We used MicroChecker V 2.2.3 [44] for stuttering and null alleles. We tested how null alleles can explain the observed *F*_IS_ using estimates of null allele frequency following either Brookfield's second method [45] or to the method of van Oosterhout et al. [44] as given by MicroCheker. We used these estimates to compute expected blank (non amplified null homozygotes) frequency assuming panmixia. For each locus, the sum of all expected blanks across subsamples was compared to the sum of all observed ones with an exact unilateral binomial test with the alternative hypothesis: there were not enough observed blank genotypes as compared to what would be expected under the hypothesis of null alleles in a panmictic population. For X linked loci we also used null allele frequencies (estimated from females) directly as the expected proportion of blank (unamplified) males expected at these loci and this quantity was also compared with observed blanks with the same method as described above for females at other loci.

Confidence intervals (CI) were obtained using the standard error of estimates obtained by jackknife over subsamples or by bootstrap over loci, using Fstat, as described in [46].

Sex-biased dispersal was assessed using three tests implemented in Fstat. First, Weir and Cockerham's estimate of *F*_ST_, was calculated separately in each sex. Next, tests based on the mean (m*AI_c_*) and the variance (v*AI_c_*) of Favre et al.'s corrected assignment index *AI_c_* [47] were performed (see Prugnolle and De Meeûs [48] for more details on these tests). All three tests are based on a permutation procedure; the sex of each individual is randomly re-assigned in each population (10,000 permutations). The observed difference between male and female *F*_ST_, the ratio of the largest to the smallest v*AI_c_* and the *AI_c_*-based *t*-statistics defined by Goudet [49] were then compared to the resulting chance distributions. For the sex that has a higher dispersal rate, *F*_ST_ and m*AI_c_* are expected to be smaller and v*AI_c_* is expected to be higher than for the sex that has a lower dispersal rate. This choice of statistics is motivated by the work of Goudet et al. [49] where v*AI_c_* was shown to be the most powerful statistic when migration is low (less than 10%), while *F*_ST_ performs better in other circumstances. We also chose to keep m*AI_c_* because it may be more powerful in case of complex patterns of sex specific genetic structures [50], [51]. Tests were all bilateral.

Isolation by distance was inferred with Rousset's procedure [52] through the regression *F*_ST_/(1-*F*_ST_)∼*a*+*b*Ln(*D_G_*). *F*_ST_/(1-*F*_ST_) is a modified measure of differentiation between two subpopulations, *a* is a constant, Ln(*D_G_*) is the natural logarithm of the geographical distance between subpopulation pairs for two dimensional data and *b* the slope of the regression that is related to the product *D_e_*σ^2^ of reproducing (effective) adults local density (*D_e_*) by the dispersal surface σ^2^ (σ is the mean distance between reproducing adults and their parents) by the equation *D_e_*σ^2^ = 1/4*πb* because the neighbourhood size *Nb* = 1/*b* = 4*πD_e_σ* ^2^ [52]. In that case, the effective number of immigrants per neighbourhood can be computed as *N_e_m* = 1/2*πb* [52]. For one dimensional data, the model becomes *F*_ST_/(1-*F*_ST_)∼*a*+*bD_G_* and *D_e_σ* ^2^ = 1/4*b* [52]. The significance of the signal was tested with a Mantel test [53] and bootstrap over loci gave 95% confidence intervals for the slope. All isolation by distance procedures were implemented using Genepop 4 [54] with 1,000,000 iterations. For the sake of power, traps were used as sub-population units for isolation by distance procedures.

Effective population sizes were estimated following Waples and Do's method based on linkage disequilibrium and implemented in LDNe [55], linkage disequilibrium and heterozygosity as implemented by Estim 1.2 [56] and following Balloux's method based on heterozygote excess in dioecious populations [57] assuming even sex ratio.

For *G. tachinoides*, since no sub-structuring was observed at the site level, areas of sites were assimilated to the rectangle defined by the approximate gallery forest width (∼100 m) and the mean maximal distance between the two most distant traps in a site (∼1000 m), being aware that it is a conservative value. This surface *S* = 100,000 m^2^ was thus used to divide effective population sizes to compute densities. For *G. p. gambiensis* densities were computed by dividing the population size by the mean minimum distance between two traps (∼100 m) in one dimension along rivers, or by the surface of the rectangle defined by this distance and the approximate gallery forest width (∼100 m), hence *S* = 10,000 m^2^, for two dimensions. This distance of 100 m also corresponds to the range of attraction of a biconical trap, and thus the smallest river section that can be sampled irrespective of the sampling protocol used [58].

## Results

### Defining the subpopulation units

HierFstat analysis only found one significant hierarchical level of population structure in the *G. tachinoides* samples, i.e. subdivision by sites *F*_Site/Basin_ = 0.026 (*P*-value = 0.001). Traps (*P*-value = 0.179) and river basin (*P*-value = 0.707) did not significantly contribute to the genetic structure of *G. tachinoides*. To check for possible disturbing effect of substructuring within sites that may not be detected by HierFstat, we also tested isolation by distance between traps in each of the four sites with the model *F*_ST_/(1-*F*_ST_)∼*a*+*bD_G_*, appropriate for one dimensional data (along the river). This analysis was feasible in view of the large amount of data available for the Mouhoun River. Absence of population sub-structuring was confirmed by the total absence of any isolation by distance between traps within the Mouhoun River (all slopes ≤0, all *P*-values>0.49). In further analyses we only considered sites as subpopulation units for *G. tachinoides*, except for isolation by distance as explained above.

For *G. p. gambiensis*, two hierarchical levels appeared to contribute significantly to genetic structure, the trap in each site (*F*_Trap/Site_ = 0.0117, *P*-value = 0.033) and the site in each river basin (*F*_Site/Basin_ = 0.0379, *P*-value = 0.001). The analysis therefore revealed that river basins were not important for the genetic structuring of the *G. p. gambiensis* populations (*P*-value>0.6). For all further analyses with *G. p. gambiensis,* the trap was considered as the subpopulation unit and, for population structure analyses (sex biased dispersal, isolation by distance), each site was considered separately, except when specified otherwise.

### Within subsamples genetic structure

For *G. tachinoides*, LD tests were carried out with all the 9 loci (36 pairs tested) and with the six most polymorphic loci, i.e. loci with no allele at frequency above or equal to 0.9 (pGp28 and pGp29 excluded, hence 21 pairs remaining). In the first case three pairs appeared in significant linkage and two pairs in the second case, which is not significantly above the 5% level in each case (binomial *P*-values are respectively 0.27 and 0.28). For *G. p. gambiensis* only one test was significant at the 5% level, which is not significantly above the proportion expected under the null hypothesis (*P*-value = 0.7628).

**Figure 2:**
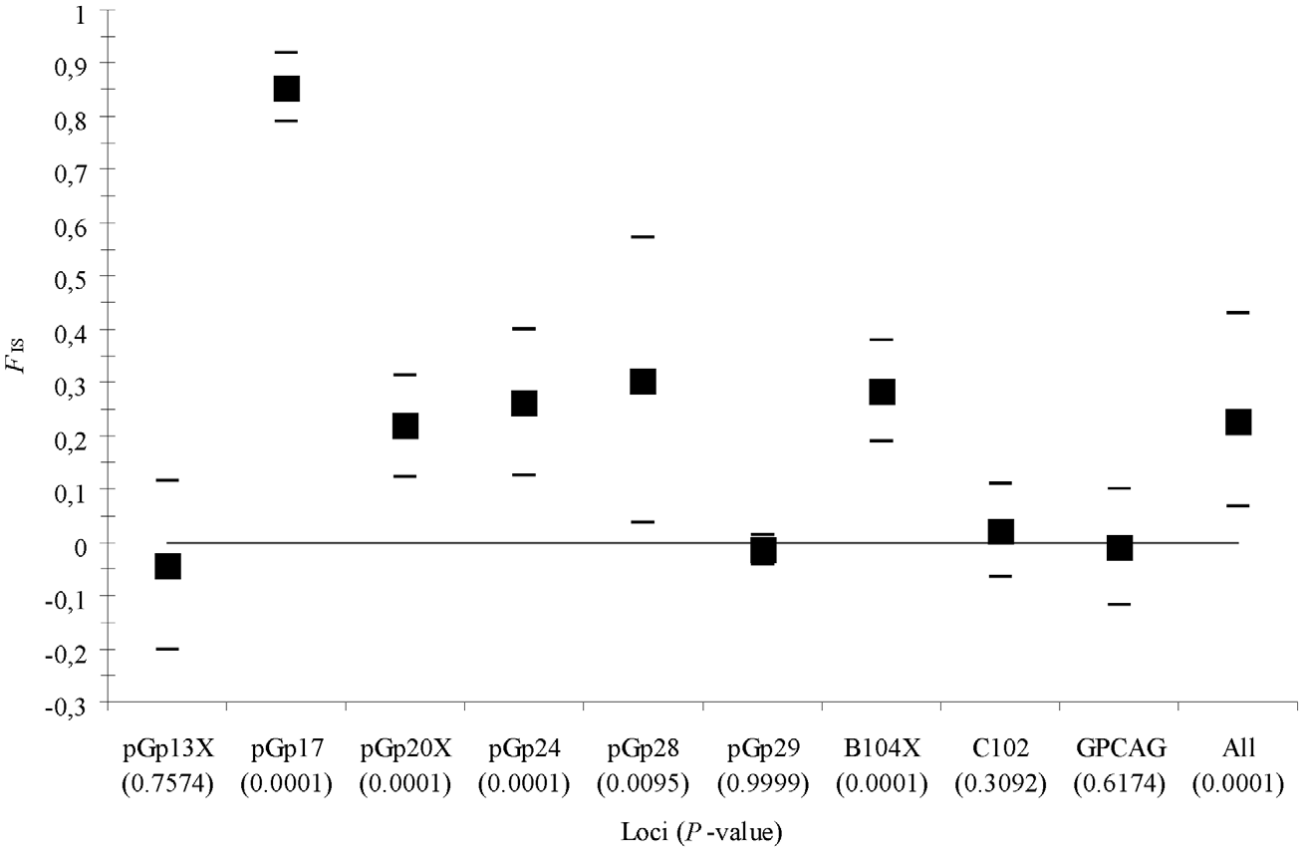
Heterozygote deficits (*F*_IS_) by locus in *G. tachinoides.* Heterozygote deficits (*F*_IS_) displayed in the different subsamples of *G. tachinoides* for each locus and over all (All). The 95% confidence intervals of each locus were obtained by jacknife over subsamples and by bootstrap over loci for the overall estimate. *P*-values, corresponding to the proportion of randomised *F*_IS_ that were above or equal to the observed *F*_IS_, are given between brackets.

There was a strong and highly significant heterozygote deficit (*F*_IS_ = 0.227, 95% CI = [0.067, 0.429] in *G. tachinoides* due to loci pGp17, pGp20X, pGp24, pGp28 and B104X (Figure 2). The four remaining loci, pGp13X, pGp29, C102 and GPCAG, together provided a pattern conforming with genotypic proportions expected under random mating: *F*_IS_ = −0.005, *P*-value = 0.5661. For the other loci, stuttering was observed for pGp17 in all the eight subsamples, and in one subsample for pGp20X. Moreover, null alleles can reasonably explain all *F*_IS_ as can be seen from Table 3. Consequently, it was assumed with confidence that stuttering and null alleles totally explained the heterozygote deficits observed at these five loci and we can confidently conclude that the *G. tachinoides* subsamples conformed to the random mating hypothesis.

**Table 3:** Null allele analyses in *G. tachinoides*.

Locus	Sex	N	Blanks	Brookfield 2	van Oosterhout	Stuttering
pGp17		296	51	76 (0.0003)	38 (0.9896)	8
pGp20X	F	170	45	39 (0.8713)	2 (1 )	1
pGp20X	M	126	33	44 (0.0233)	11 (1 )	NA
pGp24		296	137	134 (0.6378)	5 (1 )	0
pGp28		296	24	24 (0.5340)	2 (1 )	0
B104X	F	170	25	22 (0.7656)	4 (1 )	0
B104X	M	126	17	38 (0.0001)	16 (0.6758)	NA

Results are given for the loci displaying a significant departure from proportions expected under panmixia (see Figure 2). For X linked loci, results are given for females (F) and males (M). Number of genotyped individuals over all subsamples (N) and total number of blanks (Blanks) are also provided. Under random mating hypothesis, and if null alleles explain the observed heterozygote deficits, the table gives the total expected number of blank genotypes for each locus following Brookfield's second method (Brookfield 2) or van Oosterhout method. The number of subsamples where stuttering can explain in part the heterozygote deficits observed appears in the last column. Adequacy of observed blanks to expected ones is provided as an exact binomial *P*-value appearing between brackets (see text for more details).

**Figure 3:**
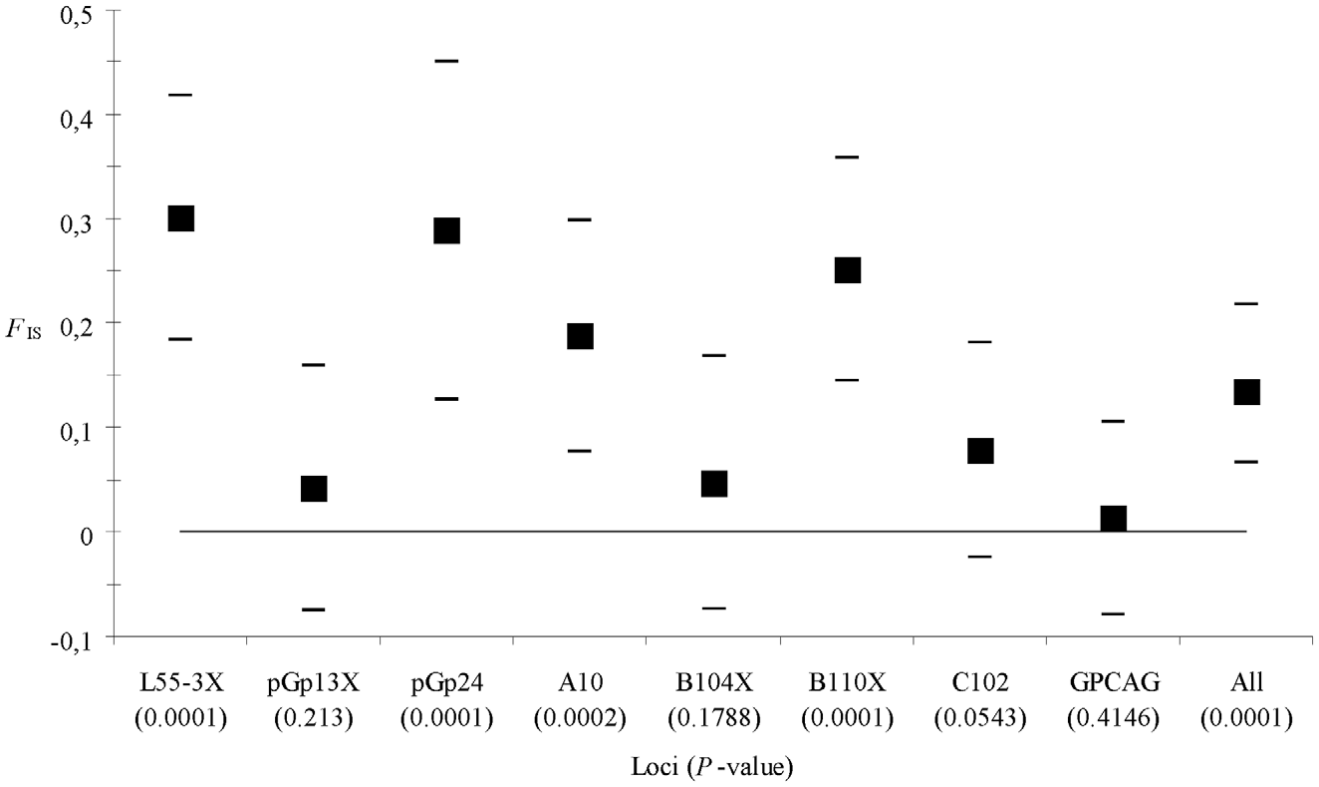
Heterozygote deficits (*F*_IS_) by locus in *G. palpalis gambiensis*. See legend of Figure 2 for details.

For *G. p. gambiensis* the *F*_IS_ is slightly lower (*F*_IS_ = 0.137, 95% CI = [0.071, 0.219]) but still highly significant (*P*-value = 0.0001) (Figure 3). According to MicroChecker analyses, null alleles provided a reasonable explanation (Table 4). Nevertheless, individually non significant loci alone still provided a significant positive *F*_IS_ = 0.042 (*P*-value = 0.0356). Thus neither null alleles nor Wahlund effects alone can explain the pattern observed in this species, as it is often the case for *G. p. gambiensis* [18], [22], [26].

**Table 4:** Null allele analyses in *G. palpalis gambiensis*. See legend of Table 3 for details.

Locus	Sex	N	Blanks	Brookfield 2	van Oosterhout	Stuttering
L55-3X	F	108	4	4 (0.5326)	3 (0.8336)	0
L55-3X	M	58	5	6 (0.5020)	7 (0.3475)	0
pGp24		166	18	17 (0.6536)	3 (1 )	0
A10		166	30	28 (0.6749)	3 (1 )	1
B110X	F	108	12	11 (0.7048)	1 (1 )	0
B110X	M	58	9	14 (0.0967)	4 (0.9946)	0

### Sex biased dispersal

As can be seen from Table 5, there is a significant genetic signature of sex biased dispersal in *G. tachinoides*, with the female flies having a lower dispersal rate (male biased dispersal).

**Table 5:** Sex biased dispersal in *G. tachinoides.*

	***F*** **_ST_**	**m** ***AI_c_***	**v** ***AI_c_***
Females	0.0417	0.24373	5.83009
Males	0.0241	−0.32884	9.53443
*P*-value	0.1494	0.0869	0.003
	***F*** **_ST_**	**m** ***AI_c_***	**v** ***AI_c_***
Females	0.0417	0.24373	5.83009
Males	0.0241	−0.32884	9.53443
*P*-value	0.1494	0.0869	0.003

Results were assessed between samples from different river basins (m*AI_c_* represents the mean and v*AI_c_* the variance of Favre et al.'s corrected assignment index *AI_c_*).

For *G. p. gambiensis* several sex biased dispersal tests were carried out:between sites over all river basins and between sites within the Mouhoun river basins, between traps within the Mouhoun river basin and between traps within sites. For the first and second tests, only one male and one female per trap were used, or only a single individual if only one sex was available, per trap and individuals of the same site considered as belonging to the same subpopulation. This data reduction was done to limit as much as possible the confounding effect of the significant differentiation that exists between traps in this species (see [51] for comments on that matter). A single test resulted in a significant *P*-value (Table 6), with the *mAI_c_* indicating a female biased dispersal. However, it can be seen from Table 6 that biased dispersal genetic signatures are inconsistent across parameters in the same analysis or across analyses for the same parameter. As previously observed [26], the most obvious conclusion, is that no genetic signature of sex biased dispersal could be detected in *G. p. gambiensis* at any level.

**Table 6:** Sex biased dispersal in *G. palpalis gambiensis.*

Analysis	*N*	Sex	*mAI_c_*	*vAI_c_*	*F*_ST_
Over all sites (1F,1M/trap, 8 sites)	27	F	−**0.507 (0.223)**	**9.932 (0.716)**	0.040
	23	M	0.595	7.337	−**0.005 (0.322)**
Over all Mouhoun (1F,1M/trap, 6 sites)	22	F	−**0.598 (0.229)**	**10.783 (0.825)**	0.031
	18	M	0.731	8.537	−**0.022 (0.310)**
Darsalamy (4 traps)	18	F	−**1.260 (0.028)**	**13.862 (0.338)**	**0.035 (0.298)**
	15	M	1.513	6.435	0.104
Minsin (2 traps)	15	F	0.123	**14.511 (0.064)**	−**0.019 (0.485)**
	8	M	−**0.231 (0.775)**	3.009	0.202
Mouhoun (18 traps)	106	F	−**0.224 (0.250)**	**11.843 (0.151)**	**0.039 (0.887)**
	72	M	0.329	7.769	0.043
RzBanson (3 traps)	13	F	−**0.343 (0.551)**	**9.320 (0.739)**	0.014
	10	M	0.447	7.642	−**0.064 (0.285)**
Samandeni (2 traps)	24	F	0.029	10.846	**0.007 (0.737)**
	12	M	−**0.059 (0.933)**	**14.989 (0.640)**	0.025
Zamakologo (3 traps)	23	F	0.254	**7.279 (0.584)**	−**0.012 (0.549)**
	12	M	−**0.487 (0.433)**	5.353	0.029

Results were assessed between traps in the Mouhoun River Basin or between sites from different river basins (m*AI_c_* represents the mean and v*AI_c_* the variance of Favre et al.'s corrected assignment index *AI_c_*). The parameter estimate of the sex with a sex biased signature is bold and followed by the *P*-value between brackets. This *P*-value is in bold when significant. As indicated in the Material and Methods section, analyses are either between sites with one female and males kept per trap or between traps in each site.

### Population structure

There was a highly significant isolation by distance across traps over the total *G. tachinoides* sampling zone (*P*-value = 0.0001) with a slope *b* = 0.015. This results in a neighbourhood size *Nb*≈67 individuals. Estim did not provide a usable effective population size. Effective population sizes were relatively convergent across Waples and Do's and Balloux's methods. With Waples and Do's method, three sites (two in Comoe and one in the Mouhoun Basin) provided outputs different from infinity, with mean *N_e_* = 99.4. Balloux's method gave *N_e_* = 100. We then assumed an effective subpopulation size of ∼100. A mean sampling surface as defined above as *S*∼0.1 km^2^, resulted in an effective population density of *D_e_* = *N_e_*/*S*≈1000 flies per km^2^. Rousset's model [52] indicated a mean dispersal per generation of around 73 m for this species, or a migration rate between neighbouring sites of *m* = 1/2*πb* = 0.11.

For *G. p. gambiensis*, there was no evidence for isolation by distance in any site along rivers. But this may be due to the very short length of river portions covered in each site. As some sites were however very distant, we further used isolation by distance in a two dimensional framework. Over the entire sampling zone, a significant isolation by distance was detected (*P*-value = 0.022) with slope *b* = 0.015 and a resulting neighbourhood size *D_e_σ* ^2^≈67 individuals identical to *G. tachinoides*. Estim provided an estimate of *N_e_* = 81 and *m* = 0.286 in one trap of the Mouhoun Basin. LDNe provided only usable values for *N_e_* in four traps of the Mouhoun Basin, with mean *N_e_* = 149. The surface defined above *S*∼0.01 km^2^ leads to an effective density of *G. p. gambiensis D_e_* = *N_e_*/*S*≈8000 (for *N_e_* = 80) or *D_e_* = 15000 (for *N_e_* = 150) *G. p. gambiensis* per km^2^ in the study area. Mean dispersal per generation is thus σ = 26 m or σ = 19 m for *N_e_* = 80 and *N_e_* = 150 respectively, corresponding to migration rates of 0.13 and 0.07 respectively (with Rousset's 1997 model in two dimensions) between neighbouring subpopulations (traps).

Using the island model of migration with even sex ratio, published by Vitalis [59], and in particular using equation 10 from his paper, we checked which parameters could lead to the sex biased dispersal observed in *G. tachinoides* and the observed difference in *F*_ST_ between female and male flies. As can be seen in Table S1, the best fit of the model parameters would indicate a very low female migration rate (less than 0.01 and most probably around 0.0001), a moderate male migration rate around 0.12 (between 0.1 and 0.15) and subpopulation sizes around 100 individuals (between 80 and 120 individuals). The number of subpopulations and the mutation rate had a small influence on the results. Thus, even if some care must be taken with these values coming from an island model of migration, parameters seem quite convergent with what was inferred from *G. tachinoides* isolation by distance population structure.

## Discussion

The population genetics data presented here suggest that the savannah area of the watershed divide between two adjacent river basins does not seem to represent a significant barrier to gene flow for the two riverine tsetse species studied. The results corroborate data from an earlier preliminary study that assessed gene flow (but without clear quantification) between three populations of *G. p. gambiensis* inhabiting two tributaries of the Mouhoun and Comoé river basins in Burkina Faso [26]. For both species, isolation by distance between sites of different river basins (or even at a micro-scale for *G. p.gambiensis*) was evidenced, without a particular role of river basins. Nevertheless, for *G. palpalis gambiensis*, dispersal along rivers (in one dimension) is still more efficient than across them (i.e. in two dimensions). During the rainy season, riverine tsetse fly species disperse in the savannah areas neighbouring the river [7], probably in search of suitable hosts, like cattle, that during that time of the year do not have to enter the riparian forests to find drinking water. It is conceivable that after some days without rain, remaining flies in the savannah areas are quickly forced to find resting sites before facing desiccation and are therefore stimulated to disperse at a higher rate. Following environmental cues such as humidity or temperature gradients, these flies will need to venture back to the closest gallery forest, that might well belong to another river basin system. Tsetse dispersal processes are complex and simple random diffusion models have often been used to capture this complexity [60]. This approach seems to be inadequate as was recently confirmed by an analysis of dispersal data of sterile male *Glossina austeni* Newstead that were released homogeneously from the air. The recapture data indicated that the sterile flies congregated in the same sites that were also preferred by their wild counterparts [61]. In addition, when riverine tsetse find themselves in unsuitable sites, they are capable of dispersing up to 2km per day to reach suitable habitats (Bouyer J., unpublished data).

The analysis presented here showed that dispersal of *G. tachinoides* across river basins was ∼3 times higher than *G. p. gambiensis*, which suggests that *G. tachinoides* flies have the ability to disperse with ease despite the severe fragmentation of the riparian gallery forests in the study area [2]. *G. p. gambiensis* dispersed less along fragmented riparian forest habitat and seemed to encounter more difficulties to disperse between the remaining fragments of this suitable habitat. The fact that genetic structuring is not correlated to geographic distance at a local scale in *G. tachinoides* [25], and the higher level of genetic structuring observed for *G. p. gambiensis* populations at the micro-scale [22] corroborate these observations. *G. tachinoides* is more xerotolerant (i.e. tolerant for dry conditions) than *G. p. gambiensis*, which could lead to a different perception of habitat borders in this species [24]. Mark-release-recapture studies carried out more than 20 years ago [7] showed that, in homogeneous, unfragmented gallery forests, the two species had a similar rate of dispersal. However, capture-mark-release-recapture data do not necessarily correlate with genetic data, as was observed in *morsitans* group flies [62], since the former is a direct measure of all kinds of dispersal including hunting dispersal, whereas the latter is an indirect measure of only reproductive dispersal. Our data imply that habitat fragmentation seems to reduce the dispersal capacity of *G. p. gambiensis* much more as compared to that of *G. tachinoides*. Similar conclusions were drawn from recent mark-release-recapture experiments in Burkina Faso, where mean dispersal coefficients of 0.3 km^2^.d^−1^ and 0.05 km^2^.d^−1^ were observed corresponding to mean square displacements of 775 m/day and 316 m/day for male *G. tachinoides* (Bouyer, J., unpublished data) and *G. p. gambiensis* [22] respectively. The much lower effective density observed for *G. tachinoides* as compared to *G. p. gambiensis* is partially related to the location of the sampling sites, which were mostly along small tributaries of the Mouhoun. These are known to be preferred sites for *G. p. gambiensis* – hence the name “spring” tsetse fly [5] – but are not favoured by *G. tachinoides*. During the entire sampling process, the mean number of flies caught per trap per day were 1.04 (s.d. 1.06) and 0.13 (s.d. 1.31) for *G. p. gambiensis* and *G. tachinoides,* respectively.

Tsetse flies are polygynous where the reproductive investment of female flies far outreaches that of the male flies. As such and according to the three main asymmetries of dispersal/philopatry costs between genders favouring biased dispersal (i.e. the resource-competition hypothesis, the local mate competition hypothesis and the inbreeding hypothesis) a sex biased dispersal in tsetse flies (should it exist) would be biased towards greater mobility of the male sex (see [47] and references therein). Our analysis of the sex biased dispersal in *G. tachinoides* suggests that female flies indeed disperse very little in fragmented riparian vegetation. This seems to suggest that female *G. tachinoides* are very conservative in their dispersal behaviour and not only remain close to “known” suitable larviposition sites in these fragmented landscapes, but are also highly philopatric i.e. they deposit their larvae close to their own place of birth. This behaviour would reduce the risk of reinvasion, as only founding females would produce offspring for a new population. This result is at variance with classical mark-release recapture experiments where females were dispersing more than males [7]. One possibility to explain our result would be a sex specific local adaptation rendering immigrant females very unlikely to survive locally. Sex based differences in dispersal were not observed for *G. p. gambiensis* in the 1980's in Burkina Faso and more recently in Guinea and Burkina Faso [18], [26]. In this case, both sexes dispersed very little, which was also reflected in a high level of structuring at a more local scale [22].

In conclusion, the data presented here, combined with those from earlier studies [26], suggest that in Burkina Faso, riverine tsetse populations from adjacent river basins are exchanging genetic material, and can therefore not be considered as biologically isolated. Therefore, potential re-invasion of flies from adjacent river basins will have to be prevented by establishing buffer zones between the Mouhoun and the other river basin(s), in the framework of the PATTEC (Pan African Tsetse and Trypanosomosis Eradication Campaign) eradication project that is presently targeting the northern part of the Mouhoun River Basin. Alternatively, the campaign should be extended to adjacent infested basins to sustain the eradication.

## Supporting Information

Table S1 **Sex biased dispersal in** ***G. tachinoides*** **.** Use of Vitalis' (2002) model to estimate possible parameters that would explain observed differences in *F*_ST_ between females and males.
(XLS)
